# Improving Emergency Department Care for Suicidality in Autism: Perspectives from Autistic Youth, Caregivers, and Clinicians

**DOI:** 10.1007/s10803-024-06364-9

**Published:** 2024-05-31

**Authors:** Paige E. Cervantes, Lawrence A. Palinkas, Greta R. Conlon, Shira Richards-Rachlin, Katherine A. Sullivan, Argelinda Baroni, Sarah M. Horwitz

**Affiliations:** 1 Department of Child and Adoelscent Psychiatry, NYU Grossman School of Medicine, New York, NY USA; 2 Herbert Wertheim School of Public Health and Human Longevity Science, University of California San Diego, San Diego, CA USA; 3 Department of Psychology, University of Alabama at Birmingham, Birmingham, AL USA; 4 Department of Psychology, St. John’s University, Queens, NY USA; 5 Child and Adolescent Psychiatry, Bellevue Hospital Center, New York, NY USA; 6 Department of Psychiatry, Virginia Commonwealth University, Richmond, VA USA

**Keywords:** Autism, Youth Suicide, Suicide Screening, Emergency Department

## Abstract

**Purpose**: Emergency department (ED) visits for suicidal ideation and self-harm are more prevalent in autistic than non-autistic youth. However, providers are typically offered insufficient guidance for addressing suicide risk in autistic youth, likely impacting confidence and care. **Methods**: In this pilot study, we conducted semi-structured interviews with 17 key members of the autism community (i.e., autistic youth with a history of suicidality, caregivers of autistic youth with a history of suicidality, autism specialist clinicians, ED clinicians) to inform the development of recommendations for modifying ED care for autistic patients, with a focus on suicide risk screening and management. **Results**: Participants reported on challenges they encountered receiving or providing care and/or recommendations for improving care. Participant perspectives were aligned, and four main categories emerged: accounting for autism features, connection and youth engagement in care, caregiver and family involvement, and service system issues. **Conclusion**: As research continues in the development of autism-specific suicide risk assessment tools and management strategies, it is essential we better equip providers to address suicide risk in autistic patients, particularly in ED settings.

Suicidal thoughts and behaviors (STBs) are highly prevalent in autistic children and adolescents, with a recent meta-analysis estimating that one in four autistic youth experience suicidal ideation and nearly one in ten attempt suicide (O’Halloran et al., 2022). In fact, autistic individuals were found to attempt and die by suicide at a rate three times greater than in the general population (Kõlves et al., 2021). Autistic youth are also nine times more likely to present to the emergency department (ED) with psychiatric concerns compared to non-autistic youth (Kalb et al., 2012). Additionally, ED visits for suicidal ideation and self-harm are both more prevalent and have more sharply increased from 2006 to 2014 in autistic than non-autistic youth (Cervantes, Brown, et al., 2023).

Unfortunately, providers across clinical settings are often unprepared to address STBs in autism. Jager-Hyman et al. (2020) surveyed clinicians primarily in community mental health and autism-specific clinics and found that they endorsed significantly lower self-efficacy screening for suicide risk in autistic compared to non-autistic patients and significantly lower acceptability of the safety plan intervention for autistic compared to non-autistic patients. Cervantes, Li, et al. (2023) expanded on these findings by examining the training, knowledge, and attitudes of mental health professionals employed in a pediatric psychiatric ED service. Despite expertise in assessing and managing mental health crises, including youth STBs, the majority of these providers did not identify autism as a suicide risk factor, half reported little to very little training in suicide risk screening and management for autistic youth, and their confidence was significantly lower providing suicide-related care to autistic compared to non-autistic patients. This lack of training and confidence is a barrier to the implementation of recommended best practices for suicide prevention in the ED (Brahmbhatt et al., 2019), likely contributing to care inequities experienced by autistic youth. Further, EDs are not typically designed to best accommodate and support autistic individuals. For instance, EDs often rely on complex verbal communication in their care delivery, include numerous sensory triggers that may be aversive, have lengthy wait times and time constraints during evaluation and intervention, and there can be significant unpredictability in what is involved in a visit (Nicholas et al., 2016; Zwaigenbaum et al., 2016).

While there is recognition of the need to modify strategies used in the general population for autistic individuals (Schwartzman et al., 2021), formal tools to support adaptation across settings, including the ED, are not yet available. In fact, a recent pilot study found that while individuals with neurodevelopmental disabilities and their therapists supported suicide risk screening, therapists reported that limited training and a lack of a valid screening measure for use with this population served as barriers to assessment (Mournet et al., 2021). Fortunately, research currently underway is focusing on adapting suicide risk screening tools and intervention practices (e.g., NCT04317118, PCORI, 2021). However, until available, providers have limited guidance for providing suicide-related care to autistic youth, likely impacting their confidence and competence assessing and intervening on suicide risk within this population.

Therefore, to better equip clinicians in a pediatric psychiatric ED service at a large public hospital in the Northeastern United States, we engaged in a pilot project to develop recommendations for modifying ED care for autistic patients, with a focus on suicide risk screening and management. We conducted semi-structured interviews with four groups of individuals: autistic youth with a history of STBs, caregivers of autistic youth with a history of STBs, autism specialist clinicians, and mental health clinicians employed in the pediatric psychiatric ED. To gather in-depth information to inform these recommendations, the current study examined perspectives on: (1) the experience of STBs in the context of autism, (2) participant experiences seeking, receiving, and/or providing suicide-related services in the context of autism, and (3) recommendations for improving mental health and suicide-specific care for autistic youth broadly as well as in the ED specifically. This is the first study to obtain perspectives from autistic youth and their caregivers about the experiences seeking and receiving suicide-related care.

## Method

### Participants

#### Autistic Youth and Caregiver Participants

Five youth and six caregiver participants were recruited from local autism and mental health clinics and via relevant listservs and websites. To be eligible for participation, caregivers must have been English-speaking and the parent or guardian of an individual on the autism spectrum with a history of STBs in childhood or adolescence. Youth participants had to be between ages 13–17 years old, have fluent verbal language and be English-speaking, have a diagnosis of autism, and have a history of STBs. If current STBs were present, the youth must have been in treatment to participate in the study. We did not require that youth and their caregivers sought care in the ED to participate. This decision was made for two reasons. First, autistic youth who experienced high levels of suicidality and their caregivers may have chosen to avoid ED treatment due to the incongruence between their needs and the ED environment (e.g., clinicians without specialized training in autism). These issues would be important to learn about when considering potential recommendations for ED care. Second, because the co-occurrence of autism and STBs is poorly addressed in all care settings, not just in EDs, we believed it to be very likely that youth and caregiver experiences in other settings and corresponding recommendations would be useful and generalizable to the ED.

#### Autism Specialist Clinician Participants

Three autism specialist clinicians were recruited from the autism clinic at an affiliated private hospital. Providers in this clinic have extensive training in autism, specialize in treating co-occurring mental health concerns, and have experience with and/or are knowledgeable about the pediatric psychiatric ED service at the public hospital through their work with patients and within previous clinical collaborations. To be eligible for participation, clinicians must have had experience treating autistic youth with STBs.

#### ED Clinician Participants

Three ED clinicians were recruited from the pediatric psychiatric ED service. We included clinicians employed directly within the setting of interest to best evaluate the feasibility of recommendations informed by youth, caregiver, and autism clinician responses for the pediatric psychiatric ED service. To be eligible for participation, these clinicians must have been employed in the setting for longer than 3 months and must have had experience assessing and managing suicide risk.

### Procedures

The qualitative interviews for the current study were conducted as part of an effort to offer ED clinician training in autism and suicidality and to evaluate a screening program for autistic youth implemented within a larger NIMH-funded initiative to improve universal suicide risk screening in EDs for all youth aged 7–17. The study was approved by the NYU Langone Health institutional review board. Interested youth, caregivers, and clinicians were first screened for eligibility and given sufficient information about this study, including that participation consisted of 20–30 min of online surveys (youth, caregivers, autism specialist clinicians) to elicit feedback about the suicide screening instrument used in the screening program and a virtual interview lasting no more than 1 h that would inform recommendations presented in the training of pediatric psychiatric ED clinicians (all groups). All caregiver and clinician participants provided consent, and youth participants provided assent. Youth and caregiver participants were compensated for their participation although clinicians were not.

Individual interviews were first conducted with youth, caregivers, and autism specialist clinicians over WebEx, a secure videoconferencing application, to gather in-depth information about their experiences and develop recommendations for the training. To better support participants, autistic youth and their caregivers were offered a list of topics to be addressed in the interview. Youth were also asked if they wanted to do the interview with a caregiver present and were given the option to complete the interview over several shorter sessions. ED clinician participants were then interviewed. This order was chosen so that targeted feedback could be elicited from ED clinicians about recommendations suggested by youth, caregivers, and autism specialist clinicians in the context of the pediatric psychiatric ED. All interviews were completed by the first author, a clinical psychologist/autism services researcher, with support from two research team members.

Semi-structured interview guides were developed for each of the four groups in consultation with a senior mental health services researcher (SH) and a clinical psychologist with extensive experience assessing and treating autistic youth and adults with co-occurring mental health diagnoses and STBs (KS). Questions were also informed by previous interview guides used in similar studies of mental health service needs of autistic individuals (e.g., Maddox et al., 2020) and the Exploration, Preparation, Implementation, Sustainment (EPIS) framework (Aarons et al., 2011). The interviews for youth and caregivers inquired about personal experiences with their or their child’s STBs (e.g., When did [STB symptoms] start? What did/does [symptoms] look like? What do you think caused [symptoms]?), experience seeking and receiving care for their or their child’s mental health concerns and STBs (e.g., Did you experience any difficulty trying to access care for your child’s symptoms?), and recommendations for care broadly and in the ED (e.g., Do you think there is anything important for clinicians to know when working with kids and teens on the autism spectrum who experience these symptoms? What are they? What about for working with autistic kids and teens with suicidality in the ED specifically?). The autism specialist clinician interview covered their experiences assessing and treating co-occurring autism and STBs (e.g., What strategies or modifications to current suicide-related care practices have you found useful in working with patients with autism and suicidality?) and their recommendations for improving the assessment and treatment of suicide risk broadly and in the ED (e.g., What would the ED need to better meet the needs of youth with autism? If a child is found at risk, what types of follow-up procedures are necessary? What challenges may be present in carrying out these procedures?). The ED clinician interview focused on their experiences addressing STBs in autistic youth (e.g., Can you describe your experience with patients on the autism spectrum with suicidality in the ED? Do procedures for assessing youth with and without autism differ in the ED? Why or why not?) and their impressions on the feasibility and utility of the recommendations presented by youth, caregivers, and autism specialist clinicians for modifying suicide-related care in their setting.

### Analysis

Interviews were transcribed in real time by two research team members, compared for accuracy, and finalized. We then analyzed transcripts using thematic content analysis (Braun & Clarke, 2006). Four transcripts, one from each group, were first reviewed by the coding team, which included one clinical psychologist/researcher, two doctoral-level psychology graduate students, and two senior mental health services researchers, to become familiar with the data, understand the content of the interviews, and document initial impressions of topics and themes. Short descriptive statements or “memos” were prepared to document initial impressions of topics and themes and their relationships, and to define the boundaries of specific codes (i.e., the inclusion and exclusion criteria for assigning a specific code; Miles & Huberman, 1994). Next, transcripts were then independently coded to condense the data into analyzable units. Segments of text ranging from a phrase to several paragraphs were assigned a priori codes (i.e., from the interview guide) or emergent codes. Codes were assigned to describe connections between categories and between categories and subcategories (i.e., axial coding; Strauss & Corbin, 1998). The first and senior author then reviewed the codes and associated interview excerpts, discussed preliminary findings, and finalized themes through an iterative process of constant comparison. Two authors, the first author and a doctoral student specializing in autism, separately coded three transcripts with the final codebook and agreement was 88%. A level of agreement ranging from 66 to 97% indicates good reliability in qualitative research (Boyatzis, 1998). All differences were reviewed and discussed until consensus was achieved.

## Results

### Participant Characteristics

#### Autistic Youth and Caregiver Participants

The six caregivers and five autistic youth participating in the study represented five youth/caregiver dyads and one caregiver participating without their child. Caregiver participants included five mothers and one grandfather. Two caregivers identified as Hispanic/Latino, two as white, one as Asian, and one did not report their race/ethnicity. Two participants endorsed having some college/an Associate’s degree, one had a Bachelor’s degree, and three had graduate degrees. One caregiver reported having an autism diagnosis. Youth participants included three females and two males aged between 13 and 17 years old (*M* = 15.80, *SD* = 1.79). Two adolescents identified as Hispanic/Latino, two identified as white, and one as more than one race. All youth participating and/or described by caregivers in this study had at least one co-occurring mental health diagnosis. Most experienced late autism diagnosis, with four receiving a diagnosis at age 12 or older and one receiving a diagnosis prior to 5 years old. Five of six experienced onset of STBs during preadolescence (i.e., between 9 and 12 years old), and suicide symptoms varied across participants (Table 1). Commonly reported contributors to suicide symptoms included interpersonal difficulties (including bullying), medication changes, lack of adequate supports, and the COVID-19 pandemic. Two of six youth received ED care for psychiatric concerns. Of the four that did not, one caregiver reported that the severity of their child’s symptoms never warranted an ED visit. Three described fears of negative consequences (e.g., impact of inappropriate placement if admitted and of interactions with untrained staff) and two endorsed limited perceived therapeutic benefit from an ED visit (e.g., lack of appropriate referrals). Two stated that they would consider taking their child to the ED if symptoms continued after they obtain comprehensive supports for their child in the community, but at the time, they felt they had yet to access less restrictive therapeutic options that may be beneficial.

**Table 1:** Youth clinical information

	Current Age	Gender	Age of Autism Diagnosis	Caregiver-Reported Mental Health Diagnoses	Age of Suicide Symptom Onset	Describe Suicide Symptoms	History of ED Care
**Caregiver A**	13	Female	13	Anxiety	Preadolescence	Suicidal ideation; Suicidal statements	No
**Caregiver B**	17	Female	7	Anxiety	Preadolescence	Suicidal ideation; Self harm	No
**Youth B**							
**Caregiver C**	13	Male	12	ADHD, ODD, history of PTSD	Adolescence	Suicidal ideation; Suicidal statements; Self harm	Yes
**Youth C**							
**Caregiver D**	15	Female	15	ADHD, MDD, Social Phobia, GAD	Preadolescence	Suicidal ideation; Suicidal statements; Self harm	Yes
**Youth D**							
**Caregiver E**	17	Male	4	Unspecified depressive disorder	Preadolescence	Suicidal ideation; Suicidal statements	No
**Youth E**							
**Caregiver F**	17	Female	13	Depression, Anxiety	Preadolescence	Suicidal ideation; Suicidal statements; Self harm; Suicide attempt	No
**Youth F**							

*ADHD = attention-deficit/hyperactivity disorder; ODD = oppositional defiant disorder; PTSD = post-traumatic stress disorder; MDD = major depressive disorder; GAD = generalized anxiety disorder

#### Clinician Participants

Autism specialist clinician participants included three female doctoral-level psychologists. They had between 9 and 15 years of experience serving autistic patients (*M* = 12.67; *SD* = 3.22), and autistic patients made up 61–80% of their current caseloads. All had experience serving several autistic patients with STBs (*M* = 15.00; *SD* = 8.66). The three ED clinician participants each served a different role in the pediatric psychiatric ED and included one psychologist (PhD), one child and adolescent psychiatrist (MD), and one social worker (MSW). They endorsed having between 3 and 7 years of experience practicing in the mental health field (*M* = 4.67, *SD* = 2.08), and between 7 months-4 years of experience in the pediatric psychiatric ED service (*M* = 2.30, *SD* = 1.71). Two reported caring for autistic youth multiple times per week in the ED, and one reported caring for autistic youth approximately once per month. All had experience providing services for autistic youth at risk for suicide. Only one autism specialist clinician endorsed formal training in suicide risk assessment in the context of autism; the five other clinicians reported learning to assess suicide risk in autism through direct experience and supervision. All six clinicians learned to manage suicide risk in autism through direct experience and supervision.

### Challenges Encountered and Recommendations for Care Improvement

All participant groups reported on challenges they encountered receiving or providing care and/or recommendations for improving care within their interviews; perspectives were aligned, and four main categories emerged: accounting for autism features, connection and youth engagement in care, caregiver/family involvement, and service system issues (Table 2). Below, we present both challenges and recommendations within each category from the perspectives of autistic youth, their caregivers, autism specialist clinicians, and ED clinicians. Table 3 presents an overview of these findings.

**Table 2:** Frequently coded themes across participant groups

	Caregivers							Youth						Autism Clinicians				ED Clinicians			
	A	B	C	D	E	F	%	B	C	D	E	F	%	G	H	I	%	J	K	L	%
**Autism: Social Communication/Social Interaction Differences**																					
*Language clarity/effective communication*		✓	✓	✓	✓	✓	83.3	✓		✓			40.0	✓	✓	✓	100	✓	✓	✓	100
*Language demands*		✓	✓		✓		50.0	✓		✓			40.0	✓	✓	✓	100				0.0
*Emotion recognition/expression and social understanding*		✓	✓				33.3	✓	✓	✓	✓	✓	100	✓	✓	✓	100	✓	✓		66.7
**Autism: Features Associated with RRBs**																					
*Predictability*		✓	✓	✓			50.0	✓	✓	✓			60.0	✓	✓	✓	100		✓		33.3
*Sensory needs*		✓		✓	✓	✓	66.7			✓			20.0	✓	✓	✓	100				0.0
*Perseveration*	✓					✓	33.3					✓	20.0		✓		33.3	✓	✓	✓	100
**Connection and Youth Engagement in Care**																					
*Lack of connection and engagement*		✓	✓	✓	✓		66.7	✓	✓	✓	✓	✓	100	✓	✓		66.7	✓			33.3
*Service delivery method*			✓	✓			33.3		✓	✓			40.0	✓		✓	66.7		✓		33.3
**Caregiver and Family Involvement**																					
*Inclusion of caregiver in care*	✓	✓	✓		✓	✓	83.3		✓				20.0	✓	✓	✓	100		✓	✓	66.7
*Caregiver and family stress*	✓			✓	✓	✓	66.7						0.0	✓	✓	✓	100	✓			33.3
**Service System Issues**																					
*Workforce capacity*	✓	✓	✓	✓	✓	✓	100	✓	✓	✓	✓	✓	100	✓	✓	✓	100	✓	✓	✓	100
*Accessibility of services*	✓	✓	✓	✓	✓	✓	100	✓					20.0		✓	✓	66.7	✓		✓	66.7
*Availability of services*	✓	✓	✓	✓	✓	✓	100	✓					20.0	✓	✓	✓	100		✓		33.3
*Affordability of services*	✓	✓	✓	✓	✓		83.3						0.0				0.0			✓	33.3
*Quality of care*		✓	✓	✓	✓		66.7						0.0				0.0				0.0
*Service intensity/consistency*	✓	✓	✓			✓	66.7	✓					20.0				0.0				0.0
*Appropriateness of placement*	✓		✓	✓	✓	✓	83.3						0.0		✓		33.3	✓	✓		66.7
*Diagnostic issues*	✓	✓	✓	✓			66.7			✓			20.0			✓	33.3	✓	✓	✓	100
*Adequacy of discharge recommendations*			✓	✓	✓	✓	66.7						0.0	✓	✓		66.7				0.0

**Table 3:** Reported challenges and recommendations across themes

**Autism: Social Communication/Social Interaction Differen**ces	
***Challenges***	- **Difficulties achieving mutual understanding**: frequent miscommunications; concern about youth misinterpretation of suicide assessment questions and provider misinterpretation of youth response; attributed to use of unclear language and lack of context or specificity
	- **High language demands**: particularly within ED time constraints and common processing speed deficits
	- **Reliance on emotion recognition and expression** in psychiatric evaluation and suicide risk management strategies
	- **Differences in nonverbal expression**: may present with a flat affect; may internalize in times of distress
	- **Emotion dysregulation** may both result from and contribute to communication difficulties
***Recommendations***	- **Optimize communication**: define terms that may be difficult; check in to confirm mutual understanding; follow up on responses to ensure accurate interpretation; be direct and concrete, avoiding open-ended questions and abstract concepts when possible
	- **Reduce language demands**: accommodate processing speed differences; use fewer words; offer alternate communication strategies; use visual supports
	- **Assist in emotion recognition/expression and social understanding**: use multi-modal teaching for suicide risk management strategies; focus on explicit indicators of symptoms; give examples of emotion words and definitions when needed; help in understanding and managing social challenges (e.g., bullying, navigating friendships)
	- **Remove demands and provide space** in cases of emotion dysregulation
**Autism: Features Associated with RRBs**	
***Challenges***	- **ED visits are often unpredictable**
	- **Sensory environment** in ED is aversive, especially problematic due to long wait times
	- **Difficulties addressing perseverative nature of STBs**
***Recommendations***	- **Accommodate sensory needs**
	- **Increase predictability**: explain rationale behind questions/procedures; introduce all providers involved in care; present what to expect for the visit; address any uncertainty from the start
**Connection and Youth Engagement in Care**	
***Challenges***	- **Difficulties connecting with and feeling understood by previous providers** may contribute to distrust and lack of engagement
	- Common mental health **service delivery methods** often not optimal for autistic population
***Recommendations***	- **Take time to develop rapport and build trust**
	- **Incorporate interests** to increase engagement
	- **Change service delivery method**: incorporate role-plays and more hands-on practice of strategies; offer more frequent breaks; use rewards; shorten session length when possible
**Caregiver and Family Involvement**	
***Challenges***	- **Provider failure to include caregivers** in assessment and intervention process
	- Provider **lack of consideration for familial stress**
	- **Challenges associated with caregiver involvement**: lack of caregiver validation or prioritization of suicide symptoms; caregiver difficulty accepting autism diagnosis; different perceptions of youth’s symptoms
***Recommendations***	- **Educate families** on autism and co-occurring mental health concerns and suicide risk
	- **Address caregiver and family stress**
	- **Thoughtfully incorporate caregivers** into their child’s care
**Service System Issues**	
***Challenges***	- **Limited workforce capacity**: providers lack training and experience with autism
	- **Frequent missed or late autism diagnosis**: attributed to both failure of previous providers to detect symptoms and to inaccessibility of diagnostic services
	- **Issues with accessibility of care**: many clinics do not accept autistic youth as patients; lengthy waitlists; failure to call caregivers back; resource-intensive requirements prior to acceptance; difficulty navigating service systems
	- **Issues with availability of care**: lack of therapeutic options for autistic youth experiencing high levels of STBs; limited availability of comprehensive supports; insufficient intensity, consistency, and quality of care offered across service systems
	- **Lack of affordable care options**
	- **Lack of appropriate placement** for autistic youth across care settings, particularly in emergency and inpatient mental health settings
	- **Lack of adequate discharge recommendations and follow-up** after an ED visit or inpatient stay
***Recommendations***	*ED-specific recommendations* - **Improve ED provider training in autism** - **Consider a patient’s full clinical picture** and provide psychoeducation to caregivers
	- **Coordinate care** among all of child’s providers to promote comprehensive support
	- **Provide targeted referrals**: confirm clinics/programs recommended accept autistic youth; follow up on referrals to ensure caregivers have resources needed to secure services
	*Broader recommendations* - **Improve training of clinicians across settings**: provide education about autism and common mental health concerns; teach communication strategies and clinical flexibility; encourage clinicians to screen autistic youth for suicide regularly, ideally by providing a specialized protocol - **Build a specialized workforce** of providers with expertise in autism to provide care and consultation
	- **Increase frequency and consistency of outpatient mental health services**
	- **Improve availability of therapeutic options** between outpatient care and psychiatric hospitalization - **Provide caregiver assistance with service navigation** and securing comprehensive supports
	- **Offer peer support** and facilitate connection to the autistic community
	- **Increase research efforts and engage autistic individuals and their families** in initiatives to address suicide risk in autism

#### Autism: Social Communication and Social Interaction Differences

An area of need frequently reported by all groups was achieving mutual understanding. Youth and caregivers reported common miscommunications between the youth and provider, leaving them feeling misinterpreted and misunderstood, which may impact willingness to disclose suicide symptoms and effectiveness of care. For instance, Youth B said: “*She didn’t really understand my thoughts. Maybe she wasn’t trained in autism, maybe I wasn’t expressing myself. I felt like she wasn’t listening to me on purpose - needless to say, I felt frustrated…*” Autism clinicians reported concerns about autistic youth interpreting questions within a screening measure or interview in a different way than intended, potentially affecting the accuracy of the suicide risk assessment. For example, Autism Clinician H said: “*For some kids that we encounter that are concrete, you can ask about these things, and they are like “No, I haven’t had these thoughts,” but that’s because they are having specific thoughts that don’t overlap with their interpretation of your questions.*” These social communication differences were also reported to affect some patients’ ability to engage in safety planning, including identifying common antecedents to STBs. ED clinicians reported related difficulties interpreting and managing suicide risk in autistic youth, as youth responses to clinician questions and probes can often be inconsistent with common responses provided by youth in the general population. Across participants, these misunderstandings and miscommunications were frequently attributed to use of unclear language and lack of context and specificity. Exacerbating communication difficulties further, participants commented on high language demands common in mental health treatment coupled with the time constraints often characteristic of ED care. Concerns were reported about autistic individuals navigating these language demands, particularly in the context of processing speed deficits and high levels of distress. For instance, Caregiver B said: “*Everything is really rushed in ERs. Anyone in crisis shouldn’t be rushed, but that’s especially true for people with autism, because it takes them a while to process emotional stuff, and if you’re asking them how they are feeling, you can’t rush them*.” Lastly, there is often a reliance on emotion recognition and expression in psychiatric evaluation and suicide risk management strategies, which can be difficult for autistic youth. Youth D had reported: “*I often don’t know how to describe what’s going on and it’s often humiliating to sit there when they are asking me what’s going on and I don’t know how to describe things.”* In addition to the barrier created by requiring verbal expression of internal states, nonverbal expression of emotion often cannot be used as an indicator of distress level, as some autistic youth were reported to present with a flat affect and to internalize even in times of significant distress. Relatedly, emotion dysregulation may both result from and contribute further to communication difficulties, creating an additional barrier to effective suicide-related care and potentially overshadowing emotional difficulties experienced by the youth.

Corresponding recommendations included: (1) Optimize communication by finding a common language to talk about STBs through defining terms that may be difficult, checking in frequently with the patient to confirm mutual understanding, and following up on responses to any suicide risk screening items to ensure accurate interpretation. It was recommended to avoid open-ended questions and abstract concepts when possible and to be direct and concrete in both assessment and intervention. (2) Reduce language demands by accommodating youth processing speed (e.g., providing additional time to respond), using fewer words, offering alternate communication strategies (e.g., writing or drawing responses rather than verbalizing them), and using visual supports to provide discrete response options and to aid in teaching more complex concepts and giving instructions. (3) Assist in emotion recognition/expression and social understanding by providing psychoeducation using multi-modal teaching on differentiating thoughts, feelings, and behaviors, particularly related to identifying antecedents to STBs, and on coping strategies and use of the safety plan. When inquiring about symptoms and experiences, it was recommended that clinicians focus on more explicit indicators, such as behaviors and physiological experiences, with reduced reliance on emotion words. When needed, clinicians should give examples of emotion words and their definitions. Few youth participants also indicated that clinician help in understanding and managing social challenges, such as bullying and navigating friendships, would be beneficial. Finally, in cases of emotion dysregulation, it was recommended that clinicians remove demands (i.e., pause the evaluation) and give autistic youth space.

#### Autism: Features Associated with Restricted, Repetitive Behaviors (RRBs)

Several challenges were noted related to RRBs. First, ED visits are often unpredictable in not only what happens within the visit but also in what may happen regarding admission decision and admission length following psychiatric evaluation. Explanations of these processes and decisions were reportedly rare, which may increase distress for autistic youth. As Autistic Youth B reported, “*The worst part of autism is when I’m in a situation where I don’t know what is going to happen. I hate not knowing what is going to happen.”* In addition, the sensory environment of the ED is often aversive and difficult to cope with, especially paired with long wait times. Again, these are important because increased distress may affect patient ability to communicate their symptoms and experiences and to engage in risk management strategies. Finally, all ED clinicians and several participants across other groups discussed difficulties addressing the perseverative nature of STBs in some autistic patients, which may decrease the effectiveness of common risk management strategies. In describing this presentation, Caregiver F stated, “*Sometimes, they are stuck. It’s like a tape that plays in their head over and over again. Like the way they get stuck on other things, they get stuck on this. Yes, they are feeling pain, not to invalidate that, but they get stuck on this as the solution. So, to help them see that there are other options is important*.” One caregiver also noted concern that answering suicide-related screening questions may cause or increase youth perseveration on these issues.

Recommendations for these challenges included: (1) Accommodate sensory needs by first understanding that autistic people may have sensory aversions that impact engagement and then addressing these aversions. Some ways to address sensory aversions included offering a low-stimulation environment to wait to be evaluated away from the general waiting room, limiting wait time for autistic patients, and providing care in an isolated room free of distractions. (2) Increase predictability by clearly explaining the rationale behind interview questions and procedures, formally introducing all providers involved in the youth’s care, presenting what to expect for the visit, ideally through use of a visual schedule, and addressing any uncertainty from the start, with particular emphasis on the admission decision. ED Clinician K stated, “*I find a lot of the distress comes from not knowing what will happen next. Like, they come in with their child, and then someone takes them to the back and the parents don’t know what’s going on necessarily. So, knowing what the steps are I think will be really useful.*”

#### Connection and Youth Engagement in Care

Most youth and caregivers reported difficulties connecting with therapists in the past and feeling as though their provider did not understand their experiences, which may lead to distrust and lack of engagement in care. Concerns were also noted about how typical mental health service delivery methods are not necessarily a good fit for autistic children and adolescents. For instance, Youth D stated, “*I don’t like to sit in an office for a while, which can be hard for a lot of therapy sessions*.”

Recommendations for these concerns included: (1) Take time to develop rapport and build trust, which are necessary for disclosure. Participants reported that clinicians should ask youth questions about their experiences, interests, and life outside of the presenting concern. For instance, when asked what ED clinicians should ask children and adolescents, Youth C reported, “*How are you and your grandpa? What do you like to do at home? Outside of here? Not just ‘Are you going to kill yourself?’ or ‘Are you depressed?’*” (2) Incorporate youth interests into the evaluation and treatment of the presenting concern to increase engagement; and (3) Change the service delivery method by incorporating role-plays and more hands-on practice of suicide risk management strategies discussed, using rewards for engagement, offering more frequent breaks, or shortening the length of the session when possible.

#### Caregiver and Family Involvement

It was noted that providers often failed to include caregivers in the assessment and intervention process, such as not considering caregiver perspectives on child’s symptoms in the evaluation and not including the caregiver as a member of the treatment plan. It was also reported that clinicians did not always consider the level of familial stress associated with having an autistic child at risk for suicide. On the other hand, some noted challenges that arose related to caregiver involvement, including lack of caregiver validation or prioritization of suicide symptoms, caregiver difficulty accepting an autism diagnosis, and different perceptions of the youth’s symptoms across caregivers and the youth, that affected suicide risk assessment and management.

Recommendations therefore included: (1) Educating families on autism and co-occurring mental health concerns and suicide risk; and (2) Addressing caregiver and family stress. Caregiver F emphasized the importance of a family-based approach stating, “*Parents are the primary caregivers of these kids, and you’ve got to help them and take care of them, because otherwise these kids have no hope.*” (3) It was also recommended that caregivers be thoughtfully incorporated into their child’s care, as they have valuable insights on their behavior across time, may increase their child’s comfort level in what is often a stressful situation during an ED evaluation, and can be an asset to suicide risk management practices, such as in implementing the child’s safety plan. For instance, Autism Specialist Clinician I stated, “*Many times, you are trying to coach and teach other caregivers and people involved in the patient’s life and care how to help them to utilize the safety plan and how to utilize their coping strategies*.”

#### Service System Issues

The systems-level challenge most widely endorsed by everyone was limited workforce capacity. Providers across care settings, and particularly in EDs, were noted to lack training and experience with autism, which impacted access to and quality of care. This was particularly true for youth often labeled as “high functioning,” where several caregiver and youth participants reported experiences where difficulties were not acknowledged or were dismissed rather than accommodated. For instance, Caregiver B stated, “*I would try to communicate about [child]’s needs and [the clinician] would say, “Well, everyone experiences that.” There was a dismissiveness and a disbelief that it is drastically different than a neurotypical experience… Even if they are ‘high functioning,’ this needs to be taken into account, because they are put in position where they are misinterpreted in negative ways, it’s assumed that they don’t have problems.”* Many caregivers and ED clinicians also reported concerns about frequent missed or late autism diagnosis, which was attributed to both failure of previous providers to detect symptoms and to the inaccessibility of autism diagnostic services and was reported to affect both understanding of youth needs and access to appropriate and comprehensive supports.

Other challenges noted included the accessibility, availability, and affordability of care. One major barrier noted by clinicians and caregivers was that many mental health clinics do not accept autistic youth as patients. ED Clinician J stated, “*Referring to clinics is actually difficult because some of them won’t accept kids with ASD… they won’t take them if autism is written in their charts anywhere, so that may mean that they get no treatment for an extended amount of time, instead of some treatment. Even if it’s not tailored, it’s better than nothing.*” Beyond these exclusions, difficulty accessing necessary care, including but not limited to mental health services, was attributed to lengthy waitlists, failure of clinics to call caregivers back, and resource-intensive requirements prior to acceptance to a clinic, such as obtaining updated diagnostic reports. Navigating the many service systems involved in supporting an autistic child to achieve comprehensive care was also noted as difficult. Specific concerns about availability included the lack of therapeutic options for autistic youth experiencing high levels of suicidal thoughts and behaviors, i.e., a care setting between weekly outpatient mental health treatment and emergency services, and the limited availability of developmentally appropriate, comprehensive supports to promote full and meaningful participation in the community for autistic youth. Further, even when services are accessed, there were problems noted with the intensity and consistency and the quality of care offered across service systems, including in schools and mental health clinics.

Additional concerns included lack of appropriate placement for autistic youth across care settings, and particularly in emergency and inpatient mental health settings where care practices are not often a good fit for autistic youth (e.g., due to high reliance on social communication). Caregivers also reported on the lack of adequate discharge recommendations and follow-up after an ED visit or inpatient stay. For instance, Caregiver D stated, *“…we didn’t get a referral, we didn’t get access to care, we didn’t get… They did give me a list. I called everyone on the list, and no one was available. They described it as a desert, and they were just like, you know, ‘Gee, our hands are tied, sorry.’”*.

In terms of recommendations, those most relevant to ED clinicians included: (1) Improving ED provider training in autism; (2) Making sure a patient’s full clinical picture is taken into account and communicated to caregivers, including providing psychoeducation on co-occurring mental health conditions and referring patients with a suspected autism diagnosis for an evaluation; (3) Coordinating care among all of the child’s providers to promote comprehensive supports, and (4) Providing targeted referrals, confirming that the clinics and programs recommended accept autistic youth as patients, and following up on those referrals to ensure caregivers have the resources needed to secure services for their child after discharge.

On a broader scale, it was recommended that we: (1) Improve the training of clinicians across settings: educating them about autism and common mental health concerns, teaching communication strategies and clinical flexibility to accommodate the unique needs of autistic youth, and encouraging clinicians to screen autistic youth for suicide regularly, ideally by providing a specialized protocol for the assessment and management of suicide risk in autism. (2) It was also recommended that we continue to build a specialized workforce of providers with expertise in autism to provide care and consultation across settings. ED clinician L discussed the value of having a specialist within the ED, stating, “*I think that we need a couple autism specialists… That’s kind of a pipe dream of mine – that there could be someone who is a guiding force. We see so many different things here. It’s so easy to lose these kids in the shuffle.”* (3) Increase not only the frequency and consistency of outpatient mental health services offered, but also the availability of therapeutic options between outpatient care and psychiatric hospitalization, such as a partial hospitalization program tailored for autistic youth. (4) Provide caregivers assistance with service navigation and securing comprehensive supports for children and adolescents, including in school and through state developmental disability and mental health systems. Comprehensive care was emphasized as needed to address challenges that may be distinct from the youth’s mental health needs but contribute to their mental health symptoms. Within this domain, many caregivers focused on the importance of daily living skills, independence, and assistance during the transition to adulthood. Several youth and caregiver participants also reported that peer support and connection to the autistic community would be helpful. For instance, Youth B stated, “*Maybe you could identify some autistic teens that are feeling this way, have some group sessions with doctors, and more autistic teens to talk about experiences together to feel like they’re not completely alone*…” A few participants also reported on the importance of increasing research efforts and engaging autistic individuals and their families in these initiatives to address suicide risk in autism.

### ED Clinicians on the Feasibility of Recommendations

Recommendations provided by youth, caregivers and autism specialists were perceived as largely useful by the three ED clinician participants, though several overlapping concerns were noted regarding feasibility. ED clinicians were concerned about implementing these modifications, particularly those related to optimizing communication, developing expectations, and including caregivers, within the time constraints of an ED visit, remembering to prioritize the modifications with the many competing priorities in psychiatric emergency care, and the amount of practice required to become skilled at implementing these recommendations, especially those related to optimizing communication across providers and autistic youth. All ED clinicians reported it would be helpful to have standardized materials to implement these modifications (e.g., a standard visual schedule, a script for contacting professionals during a crisis, an adapted safety plan). Standard ED processes were noted as barriers to minimizing wait time for autistic youth, as prioritization of patients is currently determined by an established set of guidelines, and to removing aversive sensory stimuli from the waiting room, as the child does not typically have contact with the clinical team who will inquire about diagnostic status and sensory experiences until after the waiting period in most cases. One ED clinician also reported that it would be important to identify who on the clinical team is responsible for taking the lead on ensuring these modifications are implemented, as responsibility for the patient’s wellbeing is often spread across several clinical and non-clinical providers. Next, the nature of the psychiatric emergency service as an acute care setting reportedly limits the feasibility of providing service navigation supports to ensure caregiver follow-up on their referrals and of addressing difficulties caregivers may have accepting their child has or may have an autism diagnosis. Finally, while the ED clinicians fully appreciated their role in facilitating connection to services following an ED visit, systems-level barriers are often difficult to navigate, including the limited public outpatient mental health treatment options that offer care to autistic youth, the high costs associated with private, autism-specific mental health services, and the difficulties accessing autism diagnostic services.

## Discussion

There is an urgent need to better prepare the ED workforce to address suicide risk in autism, as recent research shows that ED visits for suicidal ideation and self-harm are more prevalent in autistic compared to non-autistic youth (Cervantes, Brown, et al., 2023). To inform the development of an education session for ED clinicians, we interviewed key members of the autism community to evaluate challenges encountered and recommendations for improvement in the care of autistic youth at risk for suicide. In this pilot study, we found a high degree of consensus across autistic youth with a history of STBs, their caregivers, autism specialist clinicians, and ED clinicians regarding important areas for consideration. Importantly, areas identified as needing attention were also in line with previous research on improving ED care broadly (Nicholas et al., 2016; Zwaigenbaum et al., 2016) and on community mental health services for autistic children (Brookman-Frazee et al., 2012; Brookman-Frazee, Drahota, Brookman-Frazee et al., 2012a, b) and adults (Maddox et al., 2020).

First, all groups identified a need to account for certain characteristics of autism, namely styles of social communication and social interaction and RRBs, in the provision of care. While the strategies recommended by participants to modify care in these areas were explicit and are generally regarded as best practice in the autism field, the ED clinicians highlighted the need to practice these skills for implementation to become more fluid, particularly for optimizing communication and supporting emotion recognition and expression. It would therefore be essential to evaluate implementation and provide feedback following training in these strategies. However, it is unclear if such skills can be maintained for providers caring for autistic youth infrequently (e.g., monthly). A possible alternative would be to identify key providers to become proficient in these modifications who could preferentially evaluate autism cases when they present to the ED. In contrast, it is likely that these strategies (e.g., using direct communication, increasing predictability, offering calm, low-stimulation environments) would be beneficial for all youth in distress and thus could be applied more universally in an ED setting. Interestingly, the most common challenge related to autism features endorsed by ED clinicians was managing perseveration in the context of suicide symptoms in autistic youth, but there were limited recommendations in this area. Further, caregiver concern regarding increased perseveration following suicide risk screening is noteworthy, as research in the general population has found no evidence of iatrogenic risk, suggesting screening does not increase youth distress (Gould et al., 2005). This research should be replicated in the autism population, as reactions to screening among autistic and non-autistic youth may be distinct.

While issues related to connection and youth engagement in care were endorsed by participants in each group, this was one of only four themes highlighted as needing attention by more than half of the youth interviewed, signaling that this may be an important point for autistic youth themselves that should not be ignored. Within this area, the importance of rapport building to develop trust was highlighted most often by youth followed by incorporating interests and flexibility with the service delivery method. Given the nature of the ED and the time constraints on care, implementation may be difficult. However, particularly in the context of frequently reported negative experiences with providers in the past, failure to develop rapport, build trust, and promote engagement can hold serious implications for patient willingness to disclose suicide symptoms and thus reduce the effectiveness of suicide risk assessment and management. Therefore, these strategies should be prioritized in the ED. In contrast, caregivers and clinicians were most likely to discuss caregiver involvement and caregiver and family stress, with only one youth mentioning that further inclusion of their caregiver in an ED visit would have been beneficial. While caregivers play an essential role in suicide-related care (e.g., restricting access to means, reinforcing use of the safety plan, accessing mental health services for youth), this divergence points to the importance of balancing respect for youth autonomy and preferences with the anticipated clinical benefit of caregiver involvement in deciding the degree to which caregivers should be incorporated in care (Kerns et al., 2016).

Workforce capacity was the only area discussed by all 17 participants. This was expected, given findings of low clinician confidence assessing and managing suicide risk in autistic people (Cervantes, Li, et al., 2023; Jager-Hyman et al., 2020) and research more broadly identifying lack of autism training and knowledge as a key barrier to quality ED (Nicholas et al., 2016; Zwaigenbaum et al., 2016) and mental health (Brookman-Frazee et al., 2012; Brookman-Frazee, Drahota, Brookman-Frazee et al., 2012a, b; Maddox et al., 2020) care. Related to this issue, participants highlighted the gaps experienced by autistic youth who are verbally fluent with broadly average intellectual functioning who, when identified as autistic, are often labeled as “high functioning,” provided few to no accommodations, and feel their difficulties are often invalidated or not appreciated by providers. This is problematic, as these youth have unique needs that require attention (Alvares et al., 2020). Additionally, current research suggests that it is this group, autistic youth with average intellectual functioning and co-occurring mental health conditions, that may be more likely to experience STBs (Hirvikoski et al., 2019; Kõlves et al., 2021). Therefore, efforts are needed increase attention to these issues in the formal education of providers and in the continuing education for practicing clinicians, particularly in EDs, where visits by autistic youth with STBs are frequent. It is notable that, while the sample size was small, even the autism specialist clinicians learned to assess and manage suicide risk in autism primarily through direct experience rather than formal training.

Finally, several systems-level issues were identified that likely contribute to the onset and course of STBs. It is difficult to improve discharge recommendations following an ED visit when there is limited availability of needed supports within the service system (e.g., higher level care for youth with chronic/severe STBs, affordable outpatient mental health care for autistic youth, access to comprehensive supports). Caregivers were most concerned about these systems-level issues. Therefore, particularly within the context of reported high levels of caregiver and family stress, we not only need to address these gaps in the service system, but we more immediately need to provide formal supports for caregivers navigating this system, attempting to secure services that may reduce STBs and improve the well-being of their children. Of course, access to appropriate supports is also contingent on obtaining an accurate diagnosis, so it is essential that we address the issue of missed and late autism diagnosis discussed frequently by caregivers and clinicians in this study.

### Limitations

There are several notable limitations. This was a pilot study, and while the first to address suicide risk assessment and management practices in the ED and to include autistic youth as informants, the sample size was small and interviews were transcribed in real time. Further, all participants self-selected to engage in this research effort and may not be representative of all members of the autism community. For instance, autism specialist clinicians were recruited from one private, outpatient autism clinic; the characteristics of their patients and barriers they encounter in caring for their patients may differ from autism specialists employed in other settings. ED clinician participants were mental health professionals in a pediatric psychiatric ED service. Few specialized settings like this exist across the country. It may be that ED clinicians in general settings would have different experiences and perspectives on barriers to care and the feasibility of these recommendations. However, the expertise of these clinicians in managing suicidal crisis generally was helpful in eliciting in-depth information about how addressing suicide risk in autism was distinct. It was also clinically beneficial to recruit a targeted sample, as strategies could be tailored to the needs of this specific setting where clinician training was to take place. Youth and caregiver participants were not required to have sought ED care to participate; therefore, recommendations for the ED were sometimes made without lived experience in this setting. While this decision was made intentionally, as families of autistic youth may have chosen to avoid ED treatment for a variety of reasons relevant to this study, it may have impacted the results. All youth participating in this study were verbally fluent and had a broadly average IQ per caregiver report, and there was no formal verification of youth diagnoses. Future research will need to evaluate considerations for suicide-related care in youth with a variety of clinical presentations who undergo formal testing to improve participant characterization. Next, all youth had received or were receiving mental health treatment, so their responses may not reflect those of families who had not sought or accessed care. To participate, youth must also have had a history of STBs. As universal suicide risk screening is widely recommended (Brahmbhatt et al., 2019) and selective screening of youth with behavioral concerns is required across care settings (The Joint Commission, 2019), it would be important to obtain perspectives on suicide risk assessment and management practices of autistic youth and caregivers who have not experienced STBs. Lastly, participants across groups were primarily from an urban area in the Northeastern United States, and their perspectives may not generalize to those in other areas. However, as discussed, our findings were largely aligned with previous research conducted elsewhere (Brookman-Frazee et al., 2012; Brookman-Frazee, Drahota, Brookman-Frazee et al., 2012a, b; Maddox et al., 2020; Nicholas et al., 2016; Zwaigenbaum et al., 2016). Nevertheless, to encourage broader practice and policy change, future research with a larger and more diverse sample, including but not limited to a wider range of professionals (e.g., ED nurses) and improved representation of the autistic population, will be important to delineate widespread and systemic barriers from local and organizational challenges, as well as challenges associated with ED care for youth with psychiatric needs broadly (e.g., long wait times, issues with connection, trust, and engagement) from care for autistic youth with suicidality specifically.

## Conclusion

Despite limitations, this study was the first to focus on suicide risk assessment and management practices in the ED, interviewing key members of the autism community to develop recommendations for modifying care. In the absence of validated suicide risk assessment tools and evidence-based suicide risk management strategies specific to autism, it is essential we provide this guidance to clinicians to promote the implementation of recommended suicide-related care with autistic youth. While needed research continues in the area of autism and suicide, it will be important to update recommendations as we learn more and develop and evaluate new tools and strategies. In the development of evidence-based practices for suicide risk in autism, we will need to prioritize feasibility of implementation by non-specialized providers as well as continue to engage different members of the autism community, particularly autistic youth themselves, as perspectives and priorities may vary across groups.
